# Observation of parity-time symmetry in microwave photonics

**DOI:** 10.1038/s41377-018-0035-8

**Published:** 2018-07-18

**Authors:** Yanzhong Liu, Tengfei Hao, Wei Li, Jose Capmany, Ninghua Zhu, Ming Li

**Affiliations:** 1 State Key Laboratory on Integrated Optoelectronics, Institute of Semiconductors, Chinese Academy of Sciences, Beijing, 100083 China; 2 School of Electronic, Electrical and Communication Engineering, University of Chinese Academy of Sciences, Beijing, 100049 China; 3 Photonics Research Labs, ITEAM Research Institute, Universitat Politecnica de Valencia, Camino de Vear s/n, 46022 Valencia, Spain

## Abstract

Symmetry plays a crucial role in explorations of the laws of nature. Parity-time (PT) symmetry phenomena can lead to entirely real spectra in non-Hermitian systems, which attracts considerable attention in the fields of optics and electronics because these phenomena provide a new tool for the manipulation of oscillation modes and non-reciprocal signal transmission. A potential new field of application is microwave photonics, an interdisciplinary field in which the interaction between microwaves and optical signals is exploited. In this article, we report the experimental use of PT symmetry in an optoelectronic oscillator (OEO), a key microwave photonics system that can generate single-frequency sinusoidal signals with high spectral purity. PT symmetry is theoretically analyzed and experimentally observed in an OEO with two mutually coupled active oscillation cavities via a precise manipulation of the interplay between gain and loss in the two oscillation cavities. Stable single-frequency microwave oscillation is achieved without using any optical/electrical filters for oscillation mode selection, which is an indispensable requirement in traditional OEOs. This observation opens new avenues for signal generation and processing based on the PT symmetry principle in microwave photonics.

## Microwave photonics: exploiting PT symmetry

Parity-time (PT) symmetry, a fundamental concept that has been widely explored in quantum mechanics and optics, has now been observed and put to good use in microwave photonics. Yanzhong Liu and coworkers from Beijing and Valencia built and theoretically simulated a dual-loop optoelectronic oscillator (OEO)—a device used to convert laser energy into a radiowave or microwave signal. By adjusting the level of gain and loss in the two loops it was possible to switch the system between different phases of PT symmetry and generate a pure single-mode output at 4.0703 GHz without the need for a RF filter. Furthermore, the output was found to have very low phase noise and strong side-mode suppression. The result suggests that PT symmetry may find rich applications in microwave photonics.

## Introduction

Parity-time (PT) symmetry was first proposed in 1998 by Carl Bender and Steffan Boettcher^1^. In essence, PT symmetry exploits the fact that non-Hermitian operators can exhibit real eigenvalues associated with non-orthogonal eigenstates. By increasing the level of non-hermiticity, the symmetry can be broken after passing the so-called transition point, leading to non-real eigenvalues^1–4^. Although this concept was initially developed and extended in the field of quantum mechanics^4^, its application in this field is fundamentally limited because quantum mechanical operators are inherently Hermitian. However, this is not the case in optics^5^, in which PT symmetry can be introduced by providing gain and loss under specific conditions to photonic systems. Nearly one decade after the pioneering work by El-Ganainy et al.^5^, the feasibility of translating this quantum-inspired symmetry to the optics domain has been demonstrated in a various contributions and, specifically, in coupled optical structures^5–15^. Under specific conditions, the original Schrödinger equation and Maxwell’s theory share analogous mathematical structures^5^ and different phases of PT symmetry can be achieved by managing the gain and loss in the coupled area in a convenient manner. An experimental verification of this phenomenon in optics was reported in 2010^8^, demonstrating the fruitful utilization of this quantum-induced symmetry in optics^16^.

Based on this coupled PT-symmetric structure, which can be described by scattering matrix formalism, many new photonic devices enabled by PT symmetry have been designed and fabricated^17–29,31–34^. In particular, one of the most significant applications of PT symmetry is in cavity-mode selection, in which it has been demonstrated to be a powerful technique both in photonic (i.e., microring lasers^17–20^, whispering-gallery microcavities^24,25,33,34^) as well as electronic (i.e., active LRC circuits^26^) cavities. In whispering-gallery-mode (WGM) resonators, the nonlinear effect of doped media also plays a vital role in related research^35,36^. In particular, WGM resonators, which extend PT-symmetric optics from centimeter/meter-scale structures to on-chip micro-scale structures and from waveguides to microresonators, provide a pioneering and practicable test bed for PT symmetry in optics^25^. Use of WGM resonators naturally leads to the question of whether PT symmetry can also be applied to hybrid optical-electrical devices, such as those employed in the field of microwave photonics (MWP).

In this article, we report the experimental application of PT symmetry in an MWP system. We designed an adapted dual-loop optoelectronic oscillator (OEO), which provides a test bed for PT symmetry in a hybrid optoelectronic system. Precise control is exerted to implement the same time delay in each loop. By adjusting the gain and attenuation in each loop, different phases of PT symmetry are realized. We achieve single-mode operation at 4.0703 GHz with the side-mode suppression ratio exceeding 55 dB beyond the PT transition point without the need of selective radiofrequency (RF) filters for mode selection. Measurements indicate a phase noise level of −108 dBc/Hz at 10 kHz away from the carrier, which is a relatively low level taking into consideration the total loop length (54.75 m) of the OEO. Hence, our work provides the first proof that it is possible to manipulate and tailor MWP systems by exploiting non-Hermitian degeneracy phase transitions, paving the way for a new class of PT-symmetric MWP applications in the generation, processing, control and distribution of microwave and millimeter-wave signals.

## Results

**Fig. 1:**
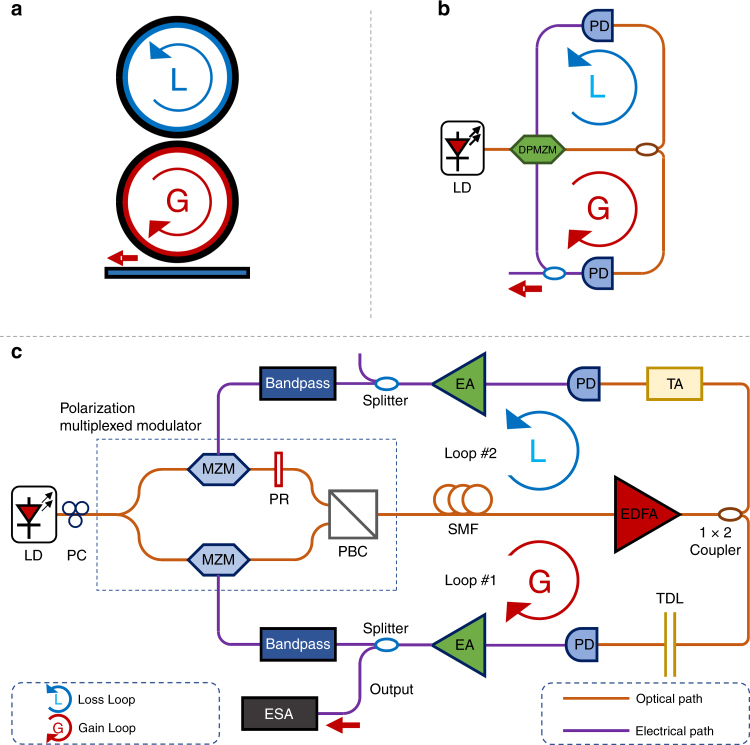
Block diagram of PT-symmetric lasers and OEOs. **a** Schematic diagram of PT-symmetric lasers. **b** Simplified schematic diagram of the adapted dual-loop OEO without high-Q filters. **c** Detailed schematic diagram of the proposed PT-symmetric OEO. LD: laser diode; PC: polarization controller; MZM: Mach-Zehnder modulator; PR: polarization rotator; PBC: polarization beam combiner; SMF: single-mode fiber; EDFA: Erbium-doped optical fiber amplifier; TA: tunable attenuator; PD: photodetector; EA: electrical amplifier; ESA: electrical spectrum analyzer; TDL: Tunable delay line

Figure 1 shows the schematic diagram of the adapted dual-loop OEO designed to study PT symmetry in an optoelectronic system. Figure 1a shows the schematic diagram of the PT-symmetric lasers. These lasers consist of two coupled microcavities. The first cavity is active and provides the gain. Meanwhile, the second cavity provides the loss. By adjusting the magnitude of the gain, loss and coupling ratio, PT symmetry can be achieved when the gain and loss are balanced to have the same magnitude. When the gain and loss are larger than the magnitude of the coupling ratio, PT symmetry will be broken and a single-mode will be selected and enhanced. Figure 1b shows a simplified schematic diagram of the adapted dual-loop OEO without high-Q filters. OEOs are typical optoelectronic hybrid oscillators that can transform continuous wave optical signals into microwave signals with high stability and high spectral purity^37^. Owing to the use of an electro-optic modulator and other effects of photonic devices, the use of OEOs is also advantageous for the generation of high-speed, and low-phase-noise signals. This type of oscillator has many practical applications in optoelectronic systems. However, this oscillator requires long cavities to achieve low phase noise operation, resulting in a very small oscillating mode frequency separation, which in turn, requires the use of very narrow-band (i.e., high-Q) RF filters and careful cavity control against environmental effects to achieve single-mode oscillation. To overcome this limitation, dual-loop OEOs^30^ have been proposed, in which a very long cavity (over several kilometers) is employed to achieve low-phase noise oscillation and a short cavity (i.e., of a few meters) is employed for single-mode selection. Although multiloop OEOs provide enhanced performance, they still require precise cavity control mechanisms to maintain the conditions for Vernier operation.

The proposed OEO is similar to a conventional dual-loop OEO, but here the two cavities have the same length (i.e., there is no mode selection cavity) and a high-Q filter is not used. An integrated dual polarization Mach-Zehnder modulator (DPMZM) is used to generate polarization multiplexed optical signals. The polarization controller makes the polarization state controllable. The DPMZM consists of two sub MZMs lying on each arm of the modulator. A polarization rotator is added to an arm to rotate the state-of-polarization of the optical signal^38,39^. A short single-mode fiber attached to the DPMZM acts as an energy storage medium. The combined use of a tunable erbium-doped optical fiber amplifier (EDFA) and tunable attenuator makes it possible to finely adjust the gain/loss in each loop of the OEO. As mentioned above, in conventional dual-loop OEOs, different loop lengths are used to take advantage of the Vernier effect for single-mode selection. However, in this PT-symmetric OEO, the cavities have equal lengths and a tunable optical delay line is inserted into one loop to ensure the same signal delay in both loops. The identical signal delay in two loops is essential for satisfying the PT symmetry condition. The longitudinal modes in each loop oscillate at the same frequency and have the same free spectral range (FSR). Although in principle, no bandpass filter is necessary to select an oscillation mode in our scheme, we incorporated wideband electrical bandpass filters from 4 to 6 GHz in each loop to eliminate low-frequency components and to conveniently observe the interaction between the two loops using a electrical spectrum analyzer (ESA). We use the two filters to ensure that the selected mode will oscillate in the desired frequency spectrum range, which is beneficial for the observation. Nevertheless, it should be noted that PT symmetry will still operate in this system even in the absence of these two filters. Two photodetectors (PDs) are used to convert the optical signal to an electrical signal^40^. In addition, electrical amplifiers (EAs) are added to boost the microwave signals in both loops.

Theoretically, the interplay between the $n$th longitudinal modes of the optical signals transmitting in the two coupled loops obey the following dynamic equations^41^ in the time domain

1 $$\frac{{da_n^{(1)}}}{{dt}} = \left( {j{\mathrm{\Delta }}\omega _n^{(1)} + g} \right)a_n^{(1)} - j\mu a_n^{(2)}$$

2 $$\frac{{da_n^{(2)}}}{{dt}} = \left( {j{\mathrm{\Delta }}\omega _n^{(2)} - \gamma } \right)a_n^{(2)} - j\mu a_n^{(1)}$$

where $a_n^{(1)}$ and $a_n^{(2)}$ represent the amplitudes of the optical signals in each loop, $\mu$ is the coupling efficient between the two loops, $\omega _n$ is the angular frequency of the $n$th longitudinal mode, and $g$ and $\gamma$ are the net gain or loss in each loop, respectively. $\omega _n^{(1,2)}$ are the resonance frequencies of each loop, and ${\mathrm{\Delta }}\omega _n^{(1,2)} = \omega _n - \omega _n^{(1,2)}$ are the optical detuning frequencies of each loop. Based on equations (1) and (2), the two supermodes of the system are given by:

3 $$\omega _{n \pm } = \frac{{\omega _n^{(1)} + \omega _n^{(2)}}}{2} + \frac{{j\left( {g - \gamma } \right)}}{2} \\ \pm \sqrt {\mu ^2 - \left( {\frac{{g + \gamma }}{2} - \frac{{j\left( {\omega _n^{(1)} - \omega _n^{(2)}} \right)}}{2}} \right)^2}$$

Assuming the same delay in the two loops, we have $\omega _n^{(1)} = \omega _n^{(2)}$. Then, under the PT symmetry condition, $g = \gamma$, Eq. (3) is simplified to

4 $$\omega _{n \pm } = \omega _n \pm \sqrt {\mu ^2 - \gamma ^2}$$

Eq. (4) reveals that the transition point is obtained when the gain/loss coefficient is equal to the coupling coefficient $\mu$. If *γ* *<* *μ*, the two cavities oscillate at slightly different real frequencies. However, when *γ* *>* *μ*, the oscillation frequency difference becomes imaginary and pairs of amplifying and decaying modes are generated in each loop.

**Fig. 2:**
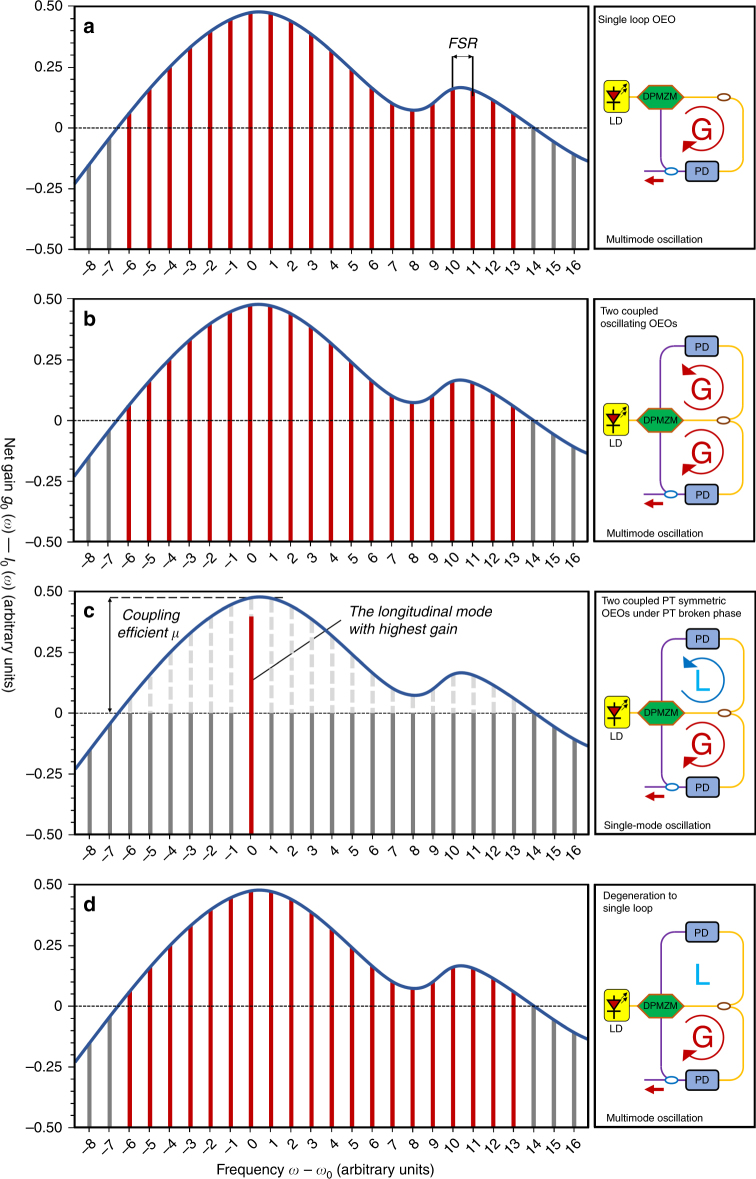
Different PT-symmetric phases according to the theory. **a** A normal single-loop OEO oscillates without a high-Q filter. All of the longitudinal modes with a positive net gain will oscillate. **b** Two coupled OEOs with same loop length oscillate without high-Q filters. All of the longitudinal modes with a positive net gain will oscillate. **c** Two coupled PT-symmetric OEOs oscillate in the PT-broken phase. The gain and loss of each loop are balanced. All of the losses overcompensate the gain for all of the longitudinal modes except for one mode by adjusting the gain and loss in the two loops. Then, a single-mode will emerge at the longitudinal mode with the highest gain, whereas other modes will be suppressed. **d** Two coupled OEOs degenerate to a single-loop OEO. The gain and loss are no longer balanced, and the loss exceeds the gain. Parity and time are no longer symmetric. The loss loop does not contribute to the oscillation

As seen in Fig. 2b, coupled loop #1 and loop #2 have the same FSR and each mode will oscillate at the same frequency. To satisfy the PT symmetry condition, the net gain in the gain loop is equal to the net loss in the loss loop

5 $$g_0 - I_0 = I_0 - \gamma _0$$

where $g_0$ is the power in the gain loop for normal oscillation, $\gamma _0$ is the power in the loss loop, and $I_0$ is the power at the critical point of oscillation.

As mentioned above, when the gain/loss exceeds the coupling coefficient $\mu$, the PT symmetry condition will be broken, the frequency eigenvalues will change from real to complex-valued and a conjugate pair of amplifying and decaying modes will appear. As in a practical OEO system, the gain profile for each mode is different, with the exception of one mode, it is possible for losses to overcompensate the gains for all of the longitudinal modes by suitably adjusting the gain and loss in the two loops. Under these conditions only a single-mode will oscillate.

Figure 2 shows different PT-symmetric phases. In our designed OEO, if the gain of the loop satisfies the oscillation condition, the OEO will oscillate. Then, multimode will emerge owing to the lack of a narrow-band filter. According to the theory, in two coupled oscillating OEOs with the same FSR, the degeneracy of each mode is broken and supermode pairs emerge. However, it is difficult to resolve mode splitting in optical fiber (i.e., non-integrated) systems^42,43^. By adjusting the gain and loss of the two loops to have equal magnitudes, each mode will oscillate under the PT symmetry condition. If we also adjust the gain and loss with enough precision to allow the longitudinal mode with the highest net gain to be larger than the coupling coefficient between the two loops, then this mode will cross the transition point to break the PT symmetry and will oscillate, while the other modes will be suppressed. Single-mode oscillation is then achieved.

**Fig. 3:**
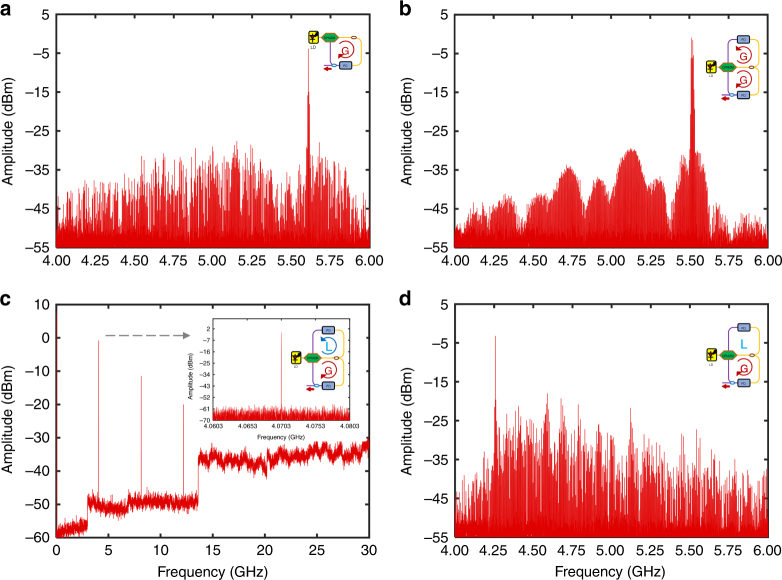
Experimental results showing the RF output in loop #1 under different conditions. **a** Emission multimode frequency spectrum with a span of 2 GHz when loop #2 is open (Resolution Bandwidth (RBW) is 100 kHz). **b** Transmission multimode frequency spectrum with a span of 2 GHz when the gain in the two loops is larger than the loss (RBW is 100 kHz). **c** Single-mode frequency spectrum appears and is stable at 4.0703 GHz with the side-mode suppression ratio exceeding 55 dB with a span of 2 GHz (RBW is 100 kHz). The modes at 8.14 GHz and 12.21 GHz are frequency multiples of the single mode caused by the nonlinear effect in the system. **d** With increasing attenuation in loop #2, the system degenerates to a single-loop with multimode from the frequency spectrum (RBW is 100 kHz)

In the experiment, we first tested the emission multimode frequency spectrum of the single gain loop. We opened loop #2 and closed loop #1. By properly adjusting the gain of loop #1 above the threshold of oscillation, loop #1 oscillates in the multimode regime because no high-Q filter is employed, as shown in Figure 3a. The optical power injected into the PD was 6.70 dBm. The threshold optical power in both loops was measured to be $I_0 = 1.13{\kern 1pt} {\mathrm{dBm}}$. Then, the loop #2 was closed, the same as loop #1. In the following steps, the gain of loop #1 was kept unchanged throughout the experiments, whereas the gain of loop #2 was tuned. By adjusting the tunable attenuator, the optical power launched to the PD in loop #2 was 2.06 dBm, which was also higher than *I*_0_. In this scenario, both loops oscillate in the multimode regime. The electrical spectrum at the output of loop #1 is illustrated in Fig. 3b. The frequency interval between the adjacent frequency components was 3.875 MHz, in good agreement with the loop length of 54.75 m. In a traditional OEO, a long single-mode fiber is necessary for energy storage. However, for this adapted PT-symmetric OEO, a long length of a single-mode fiber would lead to instability in the system due to the thermo-sensitive effect, which could disturb the observation of different PT-symmetric phases because a stable gain profile of the longitudinal modes is difficult to achieve in this case. Here, a short cavity length of 54.75 m and long cavity length of 3216 m were chosen for the experiments.

To satisfy the PT symmetry condition, as described by Eq. (5), the loss of loop #2 was calculated to be $\gamma _0 = - 4.44$ dBm. In this scenario, we successfully observed single-mode operation at 4.0703 GHz, as shown in Figure 3c. If we change the gain or loss by ~ 0.2 dB, the PT symmetry will no longer exist in the broken symmetry phase and multimode behavior will emerge, as shown in Figure 3b. It is apparent that harmonic frequency components were also generated owing to the nonlinearity of the DPMZM. We believe that the harmonic signals can be well-suppressed by replacing the DPMZM by 1 × 2 Mach-Zehnder modulator (MZM) operated in quadrature. The non-flat noise floor observed in the experimental results is a feature of the ESA that we employed; however, this is not a problem because the noise floor is flat in our observation range (4~6 GHz). Although the use of two 4~6 GHz bandpass filters limited the spectral region in which PT symmetry could be demonstrated, the filters did not influence the observation of different PT-symmetric phases and the measurements of the microwave signals. Benefitting from the PT-symmetric enhancement of single-mode gain^18^, the side-mode suppression ratio and phase noise at 10 kHz were measured to be −55 dB and −108 dBc/Hz, respectively.

Next, the loss in loop #2 was further increased to −4.94 dBm, so that the PT-broken phase condition was no longer satisfied. In this scenario, multimode operation was observed again, as shown in Figure 3d. The spectrum confirms this operation regime with a mode frequency interval of 3.875 MHz. This multimode oscillation corresponds to the single-loop oscillation shown in Figure 3a.

In an experiment, to achieve a pure PT-symmetric OEO, the signal delays in the two loops should be exactly the same to avoid the Vernier effect, which is normally employed in conventional dual-loop OEOs. Experimentally, an electrical vector network analyzer (VNA) was used to measure the path length. We first added the VNA in the electrical link of loop #2 and set loop #1 to open. We measured the loop length using the VNA. Then, we added the VNA to the electrical link of loop #1 and set loop #2 to open. Finally, we adjusted the optical tunable delay line (TDL) until the loop length matched that of the other loop. We also carried out tests to confirm that the adjustment of the passive optical tunable attenuator did not significantly change the loop length. Using the ESA, we can assure that the FSR of the modes are the same for the different operation phases; hence, no Vernier effect occurred in the cases depicted in Fig. 3a–d.

We finally excluded the possible mode selection mechanisms and focused on constructing a PT-symmetric optoelectronic system. This PT-symmetric dual-loop OEO operation makes the use of high-Q filters unnecessary, and thus, single-mode operation is achieved, as expected in an optoelectronic hybrid system. Regarding the stability, previous works on PT symmetry have mostly been based and fabricated on integrated systems with a high-Q factor transmitting optical or electrical signals. However, considering that the Q factor in our system, which is determined by the fiber length, is quite small (~ 10^3^) compared with the reported integrated PT symmetry systems in which the Q factor exceeded 10^5^ ^18,24^, the noise of the OEO signal is at a relatively low level.

## Discussion

In a traditional OEO, a high-Q electrical (or microwave photonic) filter is essential. However, no filters are required in a PT-symmetric OEO. We believe this is the key difference between our approach and the traditional one. As the PT-symmetric OEO is filter-free, the key challenge for a traditional OEO, namely, the need for a high-Q filter, is entirely avoided in our scheme.

To achieve single-mode oscillation, a single-loop with a high-Q filter is sufficient for a traditional OEO. In actual devices, high-Q filters are still a large challenge. However, a dual-loop OEO is needed for a PT-symmetric OEO, even though it is filter-free. Use of a dual-loop OEO means the system is more complicated and costly compared with the traditional approach. Thus, there is a trade-off between the PT-symmetric OEO and traditional OEO.

The PT-symmetric OEO requires dual loops to generate a single-mode oscillation. The dual-loop structure also generates the well-known Vernier effect, which also leads to a single-mode oscillation. To remove the influence of the Vernier effect, the path difference between the two loops is eliminated by precisely controlling the loop length. The coincident phase curves of the two loops are shown in Fig. S1. Related detailed methods and discussion can be achieved in supplementary materials. Unfortunately, the loop length is very sensitive to environmental changes. It is difficult to obtain two loops with the same length. It is obvious that a longer loop is more sensitive to environmental changes. Therefore, we used a short loop rather than a longer loop in our experiment to make it easier to obtain two loops with identical lengths.

Actually, the phase noise of the OEO can be easily improved using a longer loop. However, for this PT-symmetric OEO, a long single-mode fiber will cause and increase the instability of each mode owing to the thermo-sensitive effect and environmental perturbations. If a longer single-mode fiber is adopted in the OEO system for energy storage, the FSR will be much smaller. Many more multi-modes will gather in a narrow frequency band. In this case, a more precise control of the gain and loss is required.

**Fig. 4:**
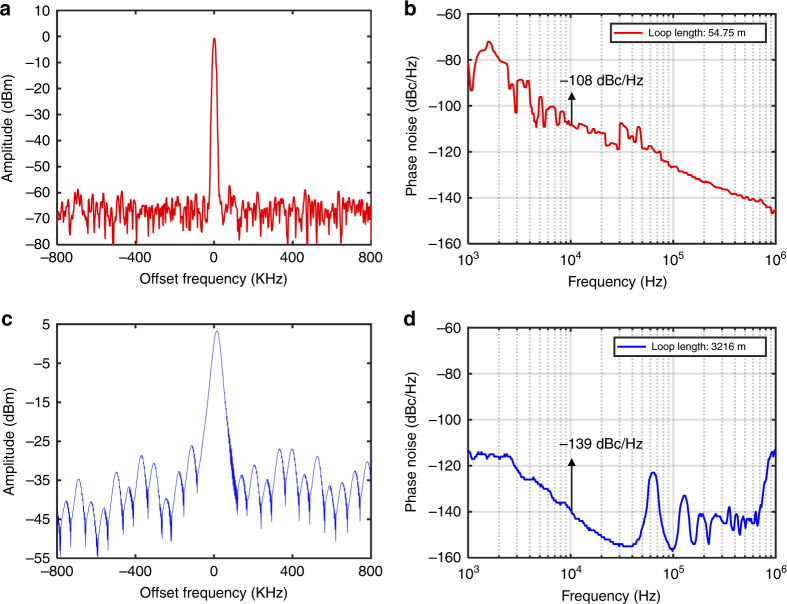
Single-mode frequency spectrum and phase noise with different loop lengths. **a** A single-mode frequency spectrum appears and is stable at 4.0703 GHz with a loop length of 54.75 m. The side-mode suppression ratio exceeds 55 dB. **b** Experimental results showing the phase noise of the oscillator. The measurement indicates that the phase noise of the system is −108 dBc/Hz at 10 kHz with a loop length of 54.75 m. **c** Single-mode frequency spectrum with a loop length of 3216 m. **d** The phase noise of the system is −139 dBc/Hz at 10 kHz with a loop length of 3216 m

To improve the phase noise of the PT-symmetric OEO, we also performed an experiment using a longer loop length of 3216 m. To remove the influence of the Vernier effect, we used a tunable delay line with a higher resolution and thermal controllers to stabilize the loops. The phase noise of the OEO was −139 dBc/Hz at the frequency offset of 10 kHz, which was 31 dB lower than the short loop case, as seen in Fig. 4d.

In conclusion, we applied PT symmetry to an important class of hybrid optoelectronic devices, the OEO. A prototype of the proposed optoelectronic hybrid system was fabricated to test the applicability of this novel concept in the MWP field. Different PT-symmetric phases of the system were observed and measured. We achieved single-mode operation in the OEO without the use of narrow-band electrical or optical filters. Single-mode operation at 4.0703 GHz with a side-mode suppression ratio exceeding 55 dB was demonstrated under the PT-symmetric broken regime. A phase noise value of −108 dBc/Hz at 10 kHz was also reported, which is a relatively good figure considering that a low loop length of 54.75 m was employed to acquire better stability conditions for the observation of PT symmetry in the system. We demonstrated that manipulation of microwaves by adjusting the gain and loss to achieve different PT-symmetric phases in an optoelectronic hybrid system was feasible and reliable. Based on this finding, the PT-symmetric OEO itself is a typical example that can be used in further applications in the MWP field. In particular, future work will explore the application of this concept to implement high-quality tunable MWP filters and pulsed RF signal generators with enhanced performance for signal processing, communication, computing, sensing, and other related fields.

## Materials and methods

The PT-symmetric dual-loop OEO in this study consisted of available commercial optoelectronic devices. An NKT Koheras Adjustik E15 laser diode provided a high output power of 18 dBm at 1550 nm. The modulator was a FTM7980EDA device produced by FUJITSU, made in Japan. We added two 90 ° hybrid couplers on each RF port to make this MZM operate as a DPMZM. The EDFA (JDS Uniphase) provided a gain of 10.6 dBm to ensure multimode oscillation. Two same HP 11982 A lightwave converters functioned as the PDs in the system. The operational range was from 1200 to 1600 nm.

The loop length measurement and the loop phase were performed by a ROHDE&SCHWARZ ZVA 40 G VNA. The output microwave frequency was measured by a ROHDE&SCHWARZ FSVR 40 GHz real-time spectrum analyzer. The phase noise was measured by a ROHDE&SCHWARZ FSWP signal analyzer.

## Electronic supplementary material

SUPPLEMENTARY MATERIAL
