# Supplementary material for: Observation of parity-time symmetry in microwave photonics
Source: Light Sci Appl. 2018 Jul 18;7:38. doi: 10.1038/s41377-018-0035-8 (PMC6107014; doi:10.1038/s41377-018-0035-8)
Supplement: Supplementary file 1 — SUPPLEMENTARY MATERIAL [file 41377_2018_35_MOESM1_ESM.docx]

**Observation of Parity–Time Symmetry in
Microwave Photonics**

**Authors**

Yanzhong Liu,^1,2^ Tengfei Hao,^1,2^ Wei Li,^1,2^ Jose Capmany^3^, Ninghua Zhu,^1,2*^ and Ming Li^1,2#^

**Affiliations**

^1^State Key Laboratory on Integrated Optoelectronics, Institute of Semiconductors, Chinese Academy of Sciences, Beijing 100083, China

^2^School of Electronic, Electrical and Communication Engineering, University of Chinese Academy of Science, Beijing 100049, China

^3^Photonics Research Labs, ITEAM Research Institute, Universitat Politecnica de Valencia, Camino de Vear s/n, 46022 Valencia, Spain

Corresponding author: [^*^nhzhu@semi.ac.cn](mailto:*nhzhu@semi.ac.cn); [^#^ml@semi.ac.cn](mailto:#ml@semi.ac.cn)

**Supplementary Materials**

1. **Optoelectronic oscillators without narrowband filters**

The principle of traditional optoelectronic oscillators (OEO) was investigated in early papers^28^. But here we focus on the principles of the OEOs without narrowband radiofrequency filters. If the electrical input signal $V_{in}\left( t \right)$ to the modulator is a sinusoidal wave with an angular frequency of $\omega$, an amplitude of $V_{0}$, and an initial phase $\beta$, then

|  | $V_{in}\left( t \right)=V_{0}\sin(\omega t+\beta)$, | (S1.1) |
| --- | --- | --- |

and the output, $V_{out}\left( t \right)$, can be obtained by expanding the left-hand side in terms of Bessel functions according to the Anger expansion formula:

|  | $V_{out}\left( t \right)=V_{ph}\{1-\eta\sin\left( \frac{\pi V_{B}}{V_{\pi}} \right)[J_{0}\left( \frac{\pi V_{0}}{V_{\pi}} \right)+2\sum_{m=1}^{\infty} J_{2m}\left( \frac{\pi V_{0}}{V_{\pi}} \right)\cos(2m\omega t+2m\beta)]-2\eta cos (\frac{\pi V_{B}}{V_{\pi}})\sum_{m=1}^{\infty} J_{2m+1}\left( \frac{\pi V_{0}}{V_{\pi}} \right)\sin[\left( 2m+1 \right)\omega t+\left( 2m+1 \right)\beta]\}$. | (S1.2) |
| --- | --- | --- |

Then we ignore the high-order harmonic components in the equation and define the voltage gain coefficient $G\left( V_{0} \right)=G_{s}\frac{2V_{\pi}}{\pi V_{0}}J_{1}(\frac{\pi V_{0}}{V_{\pi}})$. Equation S1.2 can be rewritten as:

|  | $V_{out}(t)=G\left( V_{0} \right)V_{in}(t)$. | (S1.3) |
| --- | --- | --- |

In discussion below, we rewrite equation S1.3 in complex form as

|  | ${\overset{\sim}{V}}_{out}=G\left( V_{0} \right){\overset{\sim}{V}}_{in}\left( \omega,t \right)$, | (S1.4) |
| --- | --- | --- |

where ${\overset{\sim}{V}}_{in}\left( \omega,t \right)={\overset{\sim}{V}}_{in}\left( \omega\right)\exp(\text{j}\omega t)$. Considering the signal in this OEO oscillates n times in the loop, the relation

|  | ${\overset{\sim}{V}}_{n}\left( \omega,t \right)=G\left( V_{0} \right){\overset{\sim}{V}}_{n-1}\left( \omega,t-\tau^{'} \right)$ | (S1.5) |
| --- | --- | --- |

is easy to obtain, where $\tau^{'}=\frac{n_{eff}l}{c}$ is the time delay resulting from the physical length. The initial input satisfies ${\overset{\sim}{V}}_{n=0}\left( \omega,t \right)={\overset{\sim}{V}}_{in}\left( \omega,t \right)$. Then, the total field at any instant of time can be expressed as

|  | $\overset{\sim}{V}\left( \omega,t \right)=G_{A}{\overset{\sim}{V}}_{in}\left( \omega\right)\sum_{n=0}^{\infty} \left[ G\left( V_{0} \right) \right]^{n}e^{\text{j}\omega\left( t-\tau^{'} \right)}=\frac{G_{A}{\overset{\sim}{V}}_{in}e^{\text{j}\omega t}}{1-G\left( V_{0} \right)e^{-\text{j}\omega\tau^{'}}}$. | (S1.6) |
| --- | --- | --- |

The output power of the signal is

|  | $P\left( \omega\right)=\frac{\left\vert\overset{\sim}{V}\left( \omega,t \right) \right\vert^{2}}{2R}=\frac{G_{A}\left\vert\overset{\sim}{V}\left( \omega\right) \right\vert^{2}}{2R}\frac{1}{1+\left\vert G\left( V_{0} \right) \right\vert^{2}-2\left\vert G\left( V_{0} \right) \right\vert\cos\left[ \omega\tau^{'}+\phi\left( \omega\right)+\phi_{0} \right]}$, | (S1.7) |
| --- | --- | --- |

where $\phi_{0}$ is the initial phase, $\phi\left( \omega\right)$ is phase response of the signal though different devices at the frequency of ω and R is the equivalent impedance.

(S1.7) represents a periodic train of frequency resonances that will oscillate provided that the OEO cavity gain fulfills the amplitude oscillation threshold. These resonance frequencies are given by:

|  | $\omega_{k}\tau^{'}+\phi\left( \omega_{k} \right)+\phi_{0}=2k\pi, k=0,1,2\ldots$*.* | (S1.8) |
| --- | --- | --- |

Note that the frequency separation is given by the inverse of the cavity roundtrip period, hence the longer this value the narrower is the separation between adjacent frequency modes.

1. **Parity-time symmetry equations and eigenfrequencies**

Consider now two coupled circuit loops. The coupled mode equations relating the normalized field amplitudes of the detected light signals in each loop are given by:

|  | $\frac{da_{1}}{dt}=\left( j\Delta\omega_{1}+g \right)a_{1}-j\mu a_{2}+S_{in}$, | (S2.1) |
| --- | --- | --- |
|  | $\frac{da_{2}}{dt}=\left( j\Delta\omega_{2}-\gamma\right)a_{2}-j\mu a_{1}$. | (S2.2) |

$a_{1}$, $a_{2}$ here are the normalized field amplitudes. $\Delta\omega_{1,2}=\omega-\omega_{1,2}$ is the detuning frequency where ω is the frequency of detected signal and ω_1，2_ are the oscillation frequencies of each loop. *S_in_* here is input signal to the oscillator. $\mu$ is coupling coefficient between the two loops. $g$ and $\gamma$ represent the net gain or loss in each loop respectively. We focus on the steady state oscillation regime of the device (i.e., $\frac{da_{1}}{dt}$=0, $\frac{da_{2}}{dt}=0$ and Sin=0), then:

|  | $\left( j\Delta\omega_{1}+g \right)a_{1}=j\mu a_{2}$, | (S2.3) |
| --- | --- | --- |
|  | $\left( j\Delta\omega2-\gamma\right)a_{2}=j\mu a_{1}$. | (S2.4) |

Equations S2.3 and S2.4 define a linear homogeneous system that requires a zero value for the determinant of coefficients. Thus:

|  | $\left( j\Delta\omega_{1}+g \right)\left( j\Delta\omega_{2}-\gamma\right)+\mu^{2}=0$. | (S2.5) |
| --- | --- | --- |

From this equation we get the frequency eigenvalues of the oscillator supermodes^31^:

|  | $\omega_{\pm}=\frac{\omega_{1}+\omega_{2}}{2}+\frac{j\left( g-\gamma\right)}{2}\pm\sqrt{\mu^{2}-\left( \frac{g+\gamma}{2}-\frac{j\left( \omega_{1}-\omega_{2} \right)}{2} \right)^{2}}$. | (S2.6) |
| --- | --- | --- |

In coupled PT-symmetric optical system, the gain should be equal to the loss which means $g=\gamma$. Equation S2.6 can be simplified as

|  | $\omega_{\pm}=\omega_{0}\pm\sqrt{\mu^{2}-\gamma^{2}}$. | (S2.7) |
| --- | --- | --- |

Equation (S2.7) reveals that the transition point is given by a gain/loss coefficient equal to the coupling coefficient $\mu$. If *γ<μ*, then the two cavities oscillate at slightly different real frequencies. However, when* γ>μ*, the oscillation frequency difference becomes imaginary and a pair of amplifying and decaying modes are generated in each loop respectively.

1. **Elimination of path length differences**

This PT-symmetric OEO is similar to a dual-loop OEO but using different mode selection mechanism. As the purpose of this article is to observe and study PT symmetry in Microwave Photonics, it is necessary to avoid the Vernier effect in the system. Experimentally, the time delay of each loop was precisely measured using a vector network analyzer (VNA). A tunable delay line (TDL) was inserted in loop #2 to compensate for the time difference between two loops. Moreover, this is also confirmed by the electrical delay data and Fig. 3(B) in the main text. If the Vernier effect exists, a wide band dual-loop multimode cannot be observed. The coincident phase curves of the two loops are shown in Fig. S1.

In fact, the Vernier effect generated by the path difference is also helpful to the single mode oscillation of the OEO. However, if both the Vernier effect and PT symmetry work together in the OEO, it is difficult to tell how PT symmetry selects the single mode alone.

**Figure S1 | Coincident phase curves of the two loops from 4 GHz to 6 GHz.**

1. **The single mode selection with long loop length**

We added a long single mode fiber of 3161 m in the PT-symmetric OEO and achieved the single-mode operation likewise. The whole loop length is 3216 m. Here we offer more detailed frequency spectrums with different spans. These are shown in figure S2.

**Figure S2 | Experiment results to show the single mode RF output with the loop length of 3216 m. (A)** The frequency spectrum with the span of 8 MHz. **(B)** The frequency spectrum with the span of 1.6 MHz. **(C)** The frequency spectrum with the span of 100 kHz.
